# Diagnosing Myasthenia Gravis With Repetitive Ocular Vestibular Evoked Myogenic Potentials

**DOI:** 10.3389/fneur.2020.00861

**Published:** 2020-08-13

**Authors:** Magdalena A. Wirth, Fabienne C. Fierz, Yulia Valko, Konrad P. Weber

**Affiliations:** ^1^Department of Ophthalmology, University Hospital Zurich, University of Zurich, Zurich, Switzerland; ^2^Department of Ophthalmology, University of British Columbia, Vancouver, BC, Canada; ^3^Department of Neurology, University Hospital Zurich, University of Zurich, Zurich, Switzerland

**Keywords:** myasthenia gravis diagnosis, electrophysiology, vestibular evoked myogenic potentials, repetitive nerve stimulation, ocular myasthenia

## Abstract

Timely and accurate diagnosis of myasthenia gravis, particularly in patients with fluctuating, isolated ocular involvement, remains challenging. Serological antibody testing and repetitive nerve stimulation of peripheral muscles usually have low sensitivity in these patients. Edrophonium testing may cause adverse events, single-fiber electromyography (SFEMG) is time-consuming and both tests are often unavailable outside specialized institutions. Repetitive ocular vestibular evoked myogenic potential (roVEMP) stimulation has recently been introduced to facilitate the diagnosis of myasthenia gravis. Similar to repetitive nerve stimulation, roVEMPs detect muscle decrements with the benefit of being non–invasive and allowing for direct measurement of the extraocular muscles. This review summarizes the clinical evidence of the diagnostic value of roVEMP for myasthenia. Prospective clinical trials have demonstrated high sensitivity and specificity. RoVEMPs are of particular interest in challenging myasthenia subgroups with isolated ocular involvement, negative serology, and/or negative conventional electrophysiological results. Optimal roVEMP repetition rates of 20–30 Hz have been identified. This promising novel diagnostic tool merits further attention and investigation to establish its value as a clinical test for myasthenia.

## Ocular Myasthenia Gravis – A Diagnostic Challenge

Ocular myasthenia gravis (OMG) is a rare, but potentially sinister autoimmune condition, that affects neuromuscular transmission. For various reasons extraocular muscles are particularly susceptible to transmission deficits at the neuromuscular junction, hence diplopia and/or ptosis are the initial complaint in up to 75% of patients (1). Progression to a potentially life-threatening state can occur unexpectedly. In 20–70% of patients, OMG generalizes to involve the peripheral, bulbar and/or respiratory musculature (2–4). Furthermore, myasthenia gravis can be associated with thymoma and other autoimmune conditions (5). Hence, early diagnosis and adequate treatment is of utmost importance. “Fluctuation,” the hallmark of the disease and its clinical signs, often impedes the diagnostic process. Moreover, serologic antibody testing, as well as repetitive nerve stimulation of peripheral muscles is reported to be less sensitive in OMG as compared to generalized myasthenia gravis (MG), with sensitivity rates of approximately 50% vs. up to 90% (6–12). Edrophonium testing may cause fatal adverse events and is often unavailable outside specialized institutions. With sensitivity and specificity levels above 85%, single fiber electromyography (SFEMG) of the orbicularis oculi muscle currently is the gold standard for the diagnosis of OMG, but it is time-consuming and examiner dependent (9, 13–15).

So far, several diagnostic methods, mainly using oculographic, orthoptic and tonographic parameters, have attempted to utilize eye movement fatigability for the diagnosis of OMG. For various reasons (availability, reliability, accuracy and difficulties assessing diplopia due to yoke muscle activation) none of these have been implemented in clinical practice (16). Repetitive ocular vestibular evoked myogenic potential (roVEMP) stimulation, as a non-invasive, non-pharmacological test may have the potential to fill this gap.

## Ocular Vestibular Evoked Myogenic Potentials (OVEMPS) - Overview and Clinical Utility

Ocular vestibular evoked myogenic potentials (oVEMPs) are biphasic myogenic responses to utricular stimulation representing crossed vestibulo-ocular reflexes (17). The oVEMP reflex has been shown to originate from the inferior oblique muscle and is elicited in response to otolith stimulation via bone-conducted vibration or air-conducted sound (18). After activation of the vestibular nerve and nucleus the oVEMP pathway is thought to travel through the medial longitudinal fasciculus, oculomotor nuclei and nerves to reach the extraocular muscles. They are recorded via surface electrodes from the contralateral inferior oblique muscle. In recent years oVEMPs have gained clinical significance, now forming an essential component of routine neuro-otological workup (19). Their clinical value lies in allowing for specific assessment of utricular function. OVEMPs are useful parameters for the diagnosis of diverse neuro-otological disorders e.g., Menière's disease, vestibular neuritis, vestibular Schwannoma or superior semicircular canal dehiscence (20). OVEMPs are a well-tolerated, rapid and simple diagnostic method, which can effortlessly be implemented in centers equipped for electrophysiological testing.

## Repetitive Ocular Vestibular Evoked Myogenic Potentials (ROVEMP) As A Novel Diagnostic Test for Ocular Myasthenia – A Review of The Literature

Patients with MG typically show a decrementing response to repetitive nerve stimulation. As mentioned above, in patients with isolated ocular involvement this characteristic decrement is often absent in the peripheral musculature. RoVEMP mirrors repetitive nerve stimulation, but has the key advantages of direct and non-invasive measurement of the extraocular muscles, along with exceptionally fast repetition rates.

We performed a literature search in the PubMed and Medline databases through to April 2020. The search query in PubMed was phrased as follows: (“ocular myasthenia vestibular evoked myogenic potentials” [Mesh]) OR (repetitive ocular vestibular evoked myogenic potentials^*^ [Title] AND myasthenia [Title]) OR (ocular myasthenia [Title] AND VEMP [Title]). An equipollent search query was used to search the Medline database. The references in eligible papers identified in the initial search were also screened. Four original papers of relevance were identified.

**Figure 1:**
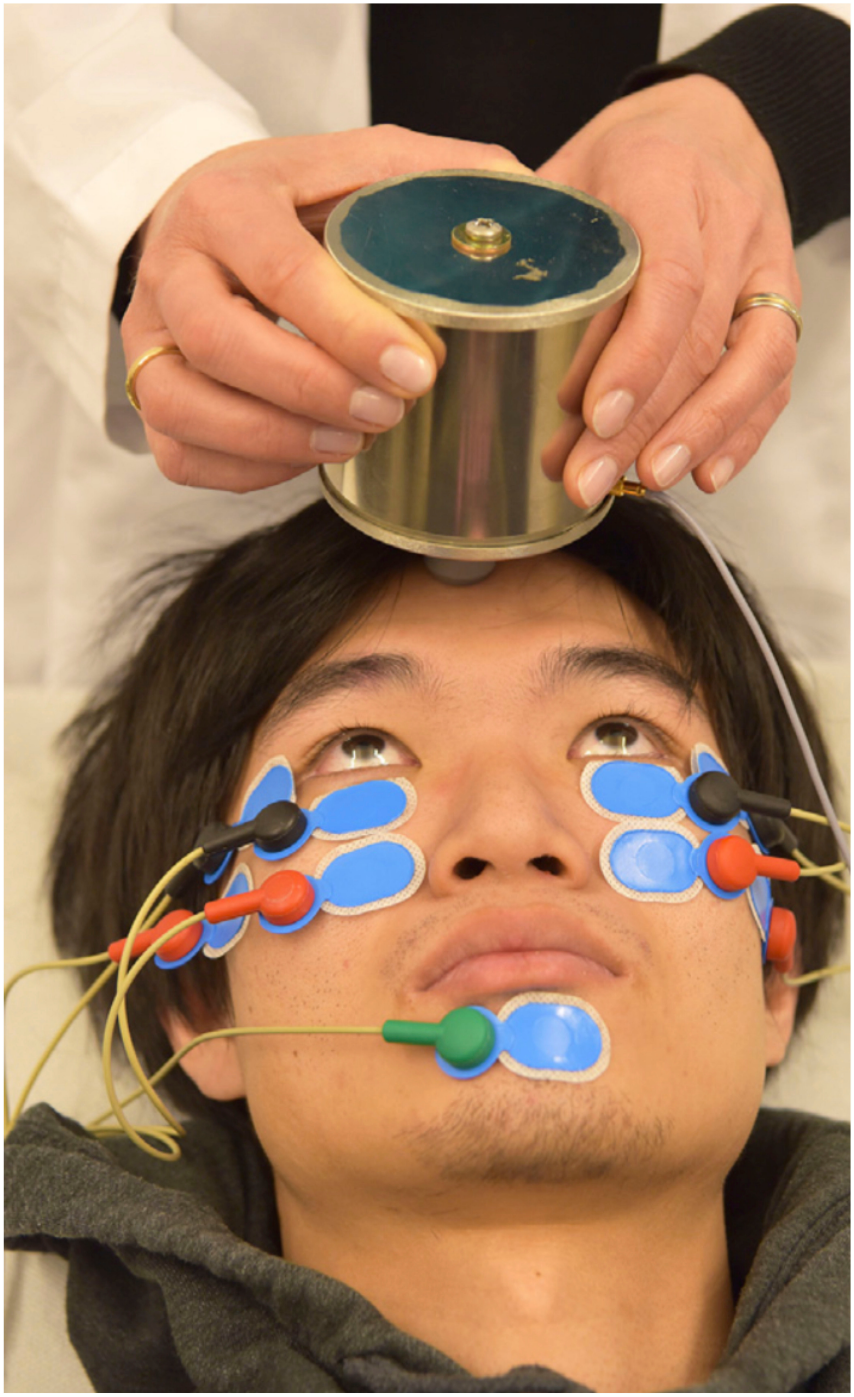
Experimental setup of roVEMP stimulation. The mini-shaker delivers bone-conducted vibration to the skull. Responses from inferior oblique extraocular muscles are recorded using surface electrodes (black: active, red: reference, green: grounding). Reprinted with permission from Wirth et al. (23).

In 2016 Valko et al. published on the first application of roVEMPs for the diagnosis of ocular myasthenia gravis. Our study included 27 myasthenic patients and 28 healthy controls. Stimulation with 4 ms bursts of 500 Hz bone-conducted skull vibration at repetition rates of 3, 10, and 20 Hz were applied, with 20 Hz yielding the most effective results. The setup for roVEMPs was similar to the standard oVEMP montage (21, 22). A train of 10 repetitive vibration bursts were delivered via a shielded hand-held mini-shaker to the forehead at the hairline in the midline (the skull location identified as standard AFz'). Responses were acquired using surface electrodes mounted at the infraorbital margins, with the reference electrodes directly below and the patient holding maximal up gaze (Figure 1 RoVEMP setup). This proof-of-concept study reported sensitivity levels of 89% when a unilateral decrement (whenever at least one of the two eyes showed a decrement) and 63% when a bilateral decrement (whenever both eyes showed a decrement) was considered. Specificity levels were 64% (for unilateral decrement) and 100% (for bilateral decrement) (24).

In 2018 El-Sayed Mojahed et al. studied whether oVEMP stimulation (without repetitive stimulation) allows for differentiation between healthy controls and various myasthenia subgroups (25). In their prospective study, the authors used air conducted oVEMP stimulation to examine a treatment naïve myasthenia group (*n* = 10), a symptomatic myasthenia group on treatment (*n* = 15) and an asymptomatic, treatment-controlled myasthenia group (*n* = 15) vs. healthy controls (*n* = 10). The authors found a significant difference of oVEMP response rate between healthy controls and myasthenia subjects (*p* = 0.002; *p* = 0.001); however no difference between the various myasthenia subgroups (*p* = 0.895) and when comparing ocular vs. generalized myasthenia patients (*p* = 0.895) was found. In conclusion, they state, that oVEMPs are a useful diagnostic parameter, yet have no value in differentiating various myasthenic subgroups or in monitoring therapeutic response.

**Figure 2:**
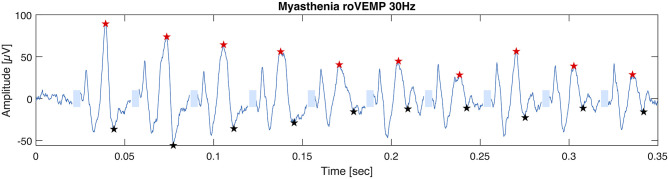
RoVEMP responses of a myasthenia subject examined with stimulus trains of 30 Hz.

**Figure 3:**
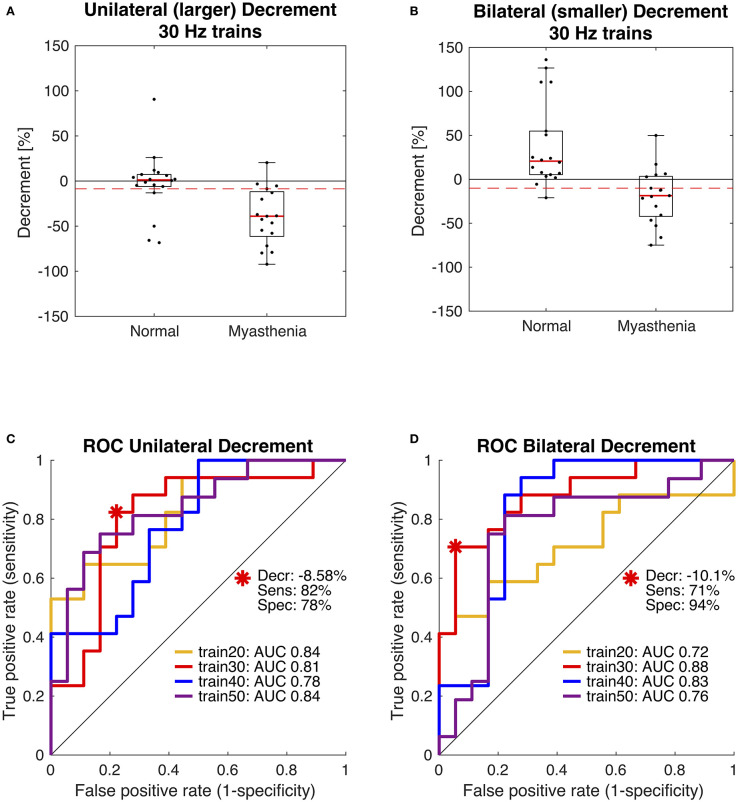
Receiver operating characteristic curve (ROC) statistics for optimal cut-off determination. Box plots in panels **(A,B)** compare the distribution of participants and show data of the 30 Hz paradigm. The dashed red lines indicate the optimal diagnostic thresholds for eyes with the larger decrement, i.e., unilateral **(A)** and for eyes with the smaller decrement, i.e., bilateral **(B)**, as derived from the red ROC curves shown in panels **(C,D)**. Area under the curve (AUC) was largest using 30 Hz trains (red). Modified and reprinted with permission from Wirth et al. (23).

In 2019 our group published another study with the purpose of optimizing the stimulation parameters of roVEMP. 18 MG patients and 20 healthy controls underwent testing for this study. A heterogeneous group of MG patients, of whom 44% reported isolated ocular symptoms, 22% bulbar weakness and 50% generalized muscle weakness, were included. Fourteen patients were on treatment at the time of testing. The experimental setup was similar to the initial description of our group in 2016 (24). We found that repetitive stimulation at 30 Hz resulted in highest sensitivity and specificity values, whereby repetition rates at 20, 40, and 50 Hz also led to a robust decrement in the inferior oblique muscles of myasthenia patients (23). (Figure 2 Single patient RoVEMP result at 30 Hz) When using the smaller decrement of the two tested eyes 30 Hz repetitive stimulation resulted in sensitivity and specificity values of 71 and 94% (area under the curve (AUC) 0.88) and 82% sensitivity and 78% specificity when considering the larger decrement for analysis (AUC 0.81) [Figure 3 Results with ROC curves of 30 Hz repetitive stimulation, modified and reprinted with permission (23)] Recordings from the inferior oblique muscle were superior to recordings from the lateral rectus muscles and continuous 100 Hz stimulation was not found to be useful for the differentiation between diseased participants and healthy controls.

A recent prospective case-control study examined whether roVEMP allows for differentiation of MG from relevant differential diagnoses, such as Lambert-Eaton myasthenic syndrome (LEMS), genetically confirmed congenital myasthenic syndrome, inclusion-body myositis, facioscapulohumeral muscular dystrophy, myotonic dystrophy, myopathy, oculopharyngeal muscular dystrophy (OPMD), chronic inflammatory demyelinating polyneuropathy, cranial nerve palsies (III, IV, VI), mechanical diplopia, and Graves' orbitopathy (GO) (26). The study included 92 MG patients, 22 healthy controls, 33 patients with a neuromuscular disease other than MG (as mentioned above), 4 LEMS patients and 2 congenital myasthenic syndrome patients. Results showed a significantly larger decrement in MG patients (28.4% ± 32.2) as compared to healthy controls (3.2% ± 13.9; *p* < 0.001) and neuromuscular controls (3.8% ± 26.9; *p* < 0.001). When considering neuromuscular controls as reference, roVEMPs resulted in a sensitivity of 67% and a specificity of 82%. The mean decrement in ocular MG (32.1% ± 23.7) and generalized MG patients (27.1% ± 34.9) was comparable. A subgroup analysis of seronegative (Acetylcholine receptor antibody) and SFEMG negative patients showed abnormal roVEMPs in 86 and 73%, respectively.

## Discussion and Future Perspectives

Current literature suggests that roVEMP may serve as a valuable, well-tolerated and inexpensive test for the diagnosis of MG. The vibration bursts used for bone-conducted oVEMP allow for stimulation at high repetition rates to drive the response of the small extraocular motor units into a decrement. Based on this unique property, roVEMP stimulation represents an ideal examination technique for detecting a decrement in extraocular muscles. This facilitates the diagnosis of seronegative and SFEMG negative ocular myasthenia, the most challenging subgroup of patients. RoVEMP stimulation has been proven useful in a number of clinical studies. Data suggest its value in distinguishing MG from other rare neuromuscular and ophthalmic diseases (i.e., LEMS, OPMD, GO etc.) and its usefulness in generalized MG.

Although usually clinically distinguishable, it is not yet clear, whether roVEMPs are also capable of differentiating other causes of ptosis (e.g., involutional/aponeurotic, congenital, ptosis in the context of Horner's syndrome) and diplopia (e.g., supranuclear palsies, mitochondrial myopathies, Duane's syndrome, strabismus etc.) from MG. Moreover, there are currently limited data about the utility of roVEMP in additional MG subgroups (e.g., patients on immunomodulatory treatment vs. treatment- naïve patients, patients post thymectomy etc.).

Further prospective studies are warranted to establish the definitive value of roVEMP in clinical practice.

## Consent Statement

Written informed consent was obtained from the individual(s) for the publication of any potentially identifiable images or data included in this article.

## Author Contributions

MW: concept and visualization, literature research, and manuscript preparation. YV and FF: literature research, manuscript revision and editing, and supplemental material. KW: supervision, concept, resources, and manuscript revision and editing. All authors contributed to the article and approved the submitted version.

## Conflict of Interest

The authors declare that the research was conducted in the absence of any commercial or financial relationships that could be construed as a potential conflict of interest.
